# Characterization of a novel mutation V136L in bone morphogenetic protein 15 identified in a woman affected by POI

**DOI:** 10.1186/s13048-021-00836-7

**Published:** 2021-06-29

**Authors:** Eleonora Ferrarini, Giuseppina De Marco, Francesca Orsolini, Elena Gianetti, Elena Benelli, Franca Fruzzetti, Tommaso Simoncini, Patrizia Agretti, Massimo Tonacchera

**Affiliations:** 1 Dipartimento Medicina Clinica E Sperimentale, Sezione Di Endocrinologia, Università Di Pisa, Via Paradisa 2, 56124 Pisa, Italy; 2 Department of Obstetrics and Gynecology, University Hospital Pisa, Pisa, Italy; 3 Laboratory of Chemistry and Endocrinology, University Hospital of Pisa, Pisa, Italy

**Keywords:** Primary ovarian failure, Mutation, Menopause, BMP-15

## Abstract

**Background:**

Premature ovarian insufficiency (POI) is an ovarian defect characterized by primary or secondary amenorrhea, hypergonadotropism and hypoestrogenism which occurs before the age of 40 years with a major genetic component. In this study we performed clinical evaluation and genetic analysis of a group of 18 patients with POI.

The study involved 18 consecutive women with POI. Karyotiping and genetic analysis for research of mutations in GDF9 (Growth Differentation Factor 9) and BMP15 (Bone morphogentic protein 15) genes and FMR1 (Fragile X Mental Retardation 1) premutation were carried out. In vitro functional study of the novel BMP15 mutation was performed using COV434 (Human ovarian granulosa tumour cells 434) cells of ovarian granulosa, which consistently express BMP responsive element, and luciferase reporter assay.

**Results:**

Three patients (17%) had a family history of POI. Ten patients (56%) had a family history of autoimmune diseases and nine patients (50%) showed a personal history of one or more autoimmune diseases. Of patients for whom morphological assessment was available, almost half (44%) had poor follicle assets or small ovaries’s size at pelvic US. Two patients (13%) showed reduced bone density at DEXA (Dual Energy X-ray Absorptiometry). All the women had normal female kariotype and no mutations in the GDF-9 gene or FMR1 premutations were found. A novel heterozygous mutation c.406G > C (V136L) of BMP15 gene was identified in one patient. After transfection in COV434 cells, BMP15 variant showed a significantly reduced luciferase activity compared to wild type.

**Conclusions:**

POI is a multifactorial disease with several health implications. Autoimmunity and genetics represent the most common aetiology. We identified and characterized a novel BMP15 mutation, providing an additional elucidation of molecular basis of this complex disorder.

## Introduction

Premature Ovariane failure (POI) is defined as a primary ovarian defect characterized by absent menarche (primary amenorrhea) or premature depletion of ovarian follicles before the age of 40 (secondary amenorrhea) with hypergonadotropism and hypoestrogenism [1].

POI affects approximately 0.01% of women aged under 20 years, 0.1% of women aged under 30 yeras and 1% women aged under 40 years [2].

The aetiology of POI is highly heterogeneous, with a wide spectrum of causes, namely cytogenetic, genetic, autoimmune, infectious or iatrogenic (chemiotherapy, radiotherapy, surgery), but often the cause remains unknown. However, it also has a major genetic component (20–25%) [3] and it is frequently found in families (4–31%) [4]. Structural and numerical abnormalities of the X chromosome, such as Turner’s syndrome and the FMR1 premutation, are the most common known congenital causes of POI [5]. The disorder usually leads to sterility and long-term deprivation of oestrogen has serious implications for female health in general, and in particular for bone, cardiovascular and neurological systems [6, 7].

Exhaustion of the pool of primordial follicles within the ovarian cortex is the cause of primary ovarian insufficiency in most women. POI may be due to two mechanisms, follicle dysfunction and follicle depletion. Factors synthesised by the oocyte or surrounding granulosa cells, such as growth differentiation factor 9, GDF9 (MIM# 601,919), and bone morphogenetic protein 15, BMP-15 (MIM# 300,247), are the primary drivers of early follicle development. They are soluble growth factors, members of the transforming growth factor β superfamily, localized on 5q.31.1 and Xp11.2 (POI region) respectively and are coexpressed in mammalian oocytes. The formation of a dominant follicle is a stepwise process that involves cell growth, proliferation and differentiation. During the early phase of this process, follicle growth and development are controlled by GDF9 and BMP-15 throughout autocrine/paracrine mechanisms [8–12].

BMP-15 heterozygotous mutations are the second in prevalence after FMR1 premutation in the pathogenesis of POI. Their frequency is variable between different ethnic groups. All the genetic variations reported in human are described at heterozygotous status and get involved the pro-region of BMP-15 gene [8].

In this study we collected clinical, biological, and genetic data related to 18 POI patients enrolled between 2009 and 2014 in our Department. We evaluated family history, clinical and/or biological data, autoimmunity, ovarian ultrasound for the presence of follicles and genetic analysis (karyotype, X chromosomal abnormalities, BMP-15, GDF-9, and FMR1 premutation).

## Materials and Methods

### Patients and controls

Eighteen consecutive women under 40 years old presenting with amenorrhea and elevated FSH (Follicle-Stimulating Hormone) levels were referred to Department of Clinical and Experimental Medicine of University of Pisa (Endocrinology section) between December 2009 and September 2014 with suspected POI. These patients were studied retrospectively.

Spontaneous POI was defined by at least 4 months of amenorrhea before 40 years old and two serum FSH levels above 25 mU/ml [13].

In our group of patients the mean age of menopause was 26.6 years. Most of our patients (n = 16; 89%) presented with normal puberty and secondary amenorrhea, while 2 patients (11%) presented with primary amenorrhea. Three patients (17%) had a family history of POI. One of these women had spontaneous pregnancy and one had a spontaneous miscarriage before the onset of POI.

One patient experienced pelvic surgery due to ovarian endometriosis cyst, treated conservatively.

No woman underwent chemioterapy or pelvic radiotherapy.

One hundred healthy women with normal ovarian function and no history of autoimmune diseases were included in the study as control.

### Hormone measurements

Serum levels of FSH and LH were measured by conventional immunoenzymatic assay (Access Immunoassay System, hLH, hFSH, BeckmanCoulter,Brea,CA, USA). Serum levels of estradiol (E2) were determined by conventional competitive immunoassay (Access Immunoassay System, Estradiol, Beckman Coulter, Brea, CA, USA). The normal range was based on hormonal results obtained in healthy women during a normal menstrual cycle and provided by the laboratory of hormonal investigations at Department of Endocrinology, Pisa. Free-T4 (fT4), free-T3 (fT3) and TSH were measured by chemiluminescent immunometric assay (Immulite 2000 Immunoassay System) at Department of Endocrinology, Pisa.

### Screening for autoimmunity

Anti-adrenal antibodies were tested by radioimmunoassay (CIS Bio International, Yvette Cedex, France). Anti-ovarian antibodies were tested for by an indirect immunofluorescence test system supplied by Biorad (Lubex) Binding Site. The measurement of antitissue transglutaminase (tTG) IgG antibodies in human serum was based on recombinant human tissue transglutaminase (Phadia US Inc., MI, USA). Anti-thyroid antibodies (anti-thyroperoxydase (AbTPO) and anti-thyroglobulin (AbTG) antibodies) were tested by a two-step immunoenzymometric assay that is performed entirely in the AIAPACK test cup (Torol Corp., Tokyo, Japan).

### Pelvic ultrasonography

Pelvic ultrasonography was performed using an Esaote sonograph and a 6.5 MHz probe by the same operator.

### BMD assessment

Femoral neck and lumbar spine BMD were assessed using DEXA (QDR-4500 Hologic Inc., Waltham, MA). BMD results at the femoral neck and lumbar spine (L1–L4) were evaluated and expressed as absolute values in g/cm2, T-scores ((BMD—peak bone mass) / s.d.) and Z-score. In accordance with World Health Organization criteria, in this cohort of young women (age < 40 years) reduced bone density was defined as a Z-score <—2.0 s.d. Normal bone has a BMD Z-score of > – 2.0 s.d. or higher.

### Genetic analysis

Genomic DNA was extracted from 400-microl peripheral lymphocytes by using EUROGOLD Blood DNA Mini Kit PLUS (EuroClone, Italy) according to the manufacturer’s instructions. Genetic analysis of GDF9, BMP-15 and FMR1 premutation was performed. Specific primers were PCR amplified and sequenced of the human BMP15 and GDF9 exons as described previously [9].

### In silico analysis to prediction of the disease-causing potential of a BMP-15 sequence variation

To predict the effect of the identified missense variant we utilized two different bioinformatics tools. These algorithmic programs were: SIFT-Sorting Intolerant From Tolerant [https://sift.bii.a-star.edu.sg/www/SIFT_seq_submit2.html] [14, 15] and PolyPhen-2-Polymorphism Phenotyping v2 [http://genetics.bwh.harvard.edu/pph2/] [16]. The variant predicted deleterious by above in silico tools was considered high risk nsSNP and investigated further.

### Construction and expression of the mutated gene

The wild type BMP-15 (wt-BMP15) gene was expressed in the vector pCS2 +  + as already described [17]. The new variant of the human BMP-15 (V136L) identified in a patient affected by POI was obtained by site-directed mutagenesis using the QuickChange Site-Directed Mutagenesis Kit (Agilent Technologies) and specific couples of primers into the pCS2 +  + . The accuracy of the recombinant construct was verified by direct sequencing.

### Cell cultures and in vitro functional assay

The human ovarian granulosa cell line COV434 were purchase from Sigma (ECACC 07,071,909) and maintained in DMEM supplemented with 2 mM L-glutamine, 25 mM D-Glucosio, 50 U/mL Penicilline, 50 microgr/mL Streptomycin and 10% FBS (Sigma, Italy) and plated in 12-well plate (2 × 10^5^ cells for well). One day after seeding, cells were cotransfected with wild-type or mutant BMP15 (500 ngr/well) and the BRE-luciferase reporter (100 ngr/well) by using Fugene 6 Reagent, following the manufactur’s instructions (Roche Diagnostics Corporation, USA). To perform the assay a pGL3 BRE-luciferase reporter vector (Addgene, USA, plasmid # 45,126) [18] and the pCS2 +  + empty vector as negative control were used. The plasmid pRL-TK (Promega, USA) containing the Renilla luciferase gene was used as internal control (10 ngr/well) during the cotransfection assays. After 24 h transfection, cells were fed fresh culture medium and only in positive control wells the medium was replaced with 1% serum medium with 10 ngr/ml of rhBMP-4 (R&D Systems) added [19].

After treating for 16 h, cell lysates were analyzed with the Dual Luciferase Reporter Assay system (Promega, USA) [19]. Luminescence in relative light units (RLU) was measured for 10 s in a Lumat 3 Instrument (Berthold Techonologies, Germany). Relative luciferase expression was determined as the ratio of Firefly to Renilla luciferase activity, Firefly luciferase activity was normalized and was expressed relative to wild type (set to 100%).

### Statistics

Results were expressed as mean ± SD of triplicate in 3 separate experiments. Differences between groups were determined using Student’s *t-test.* Data were considered statistically significant at *p-value* < 0.05.

## Results

### Hormonal evaluation

All the patients presented FSH serum levels > 25 mU/ml. The mean FSH serum level was 94.8 ± 40.8 mU/ml and the mean LH level was 41.3 ± 18.9 mU/ml. The mean E2 serum level was 26.7 ± 15.9 pg/ml. All patients were euthyroid, with fT3, fT4 and TSH serum levels in the normal range. The clinical characterisitcs of the patients and controls are summarized in Table 1.

**Table 1:** Clinical characteristics of women with primary ovarian insufficiency (POI) and controls

Characteristic	POI (*n* = 18)	Controls (*n* = 100)
Luteinizing hormone (mU/mL)	41,3 ± 18,9	9,4 ± 7,2
Follicle stimulating hormone (mU/mL)	94,8 ± 40,8	6,3 ± 2,4
Estradiol (pg/mL)	26,7 ± 15,9	68,5 ± 43,1

### Autoimmunity

Ten patients (56%) had a family history of autoimmune diseases (diabetes mellitus, autoimmune thyroiditis, vitiligo).

Nine patients (50%) showed a personal history of one or more autoimmune diseases: two patients presented Graves’ disease, seven patients had autoimmune thyroiditis. One patient had celiac disease and one had an history of diffused giant urticaria.

One patients had anti-ovarian theca antibodies.

Antithyreoglobuline antibodies were positive in seven patients and in three of them antithyreoperoxidase antibodies were positive too.

One patients showed anti gastric parietal cells antibodies.

### Pelvic ultrasonography

16/18 patients underwent transvaginal pelvic ultrasonography for evaluation of uterus and ovary’s size and structure and follicle’s presence. Six of these patients had poor follicle assets, characterized by few millimetric follicles. In 1 patient ovaries were badly observed because of their small sizes.

### BMD assessment

15/18 patients underwent femoral and lumbar spine osteodensitometry. 2 patients showed reduced bone density (Z-score < -2.0). Mean lumbar spine Z-score was – 0,58; mean femoral Z-score was – 0,6.

### Genetic analysis

The analysis of the kariotype of the whole cohort of women did not show any abnormality (46 XX).

There weren’t any abnormalities of the X chromosome such as X monosomy (Turner’s syndrome), X deletions, chromosome X translocations.

**Fig. 1:**
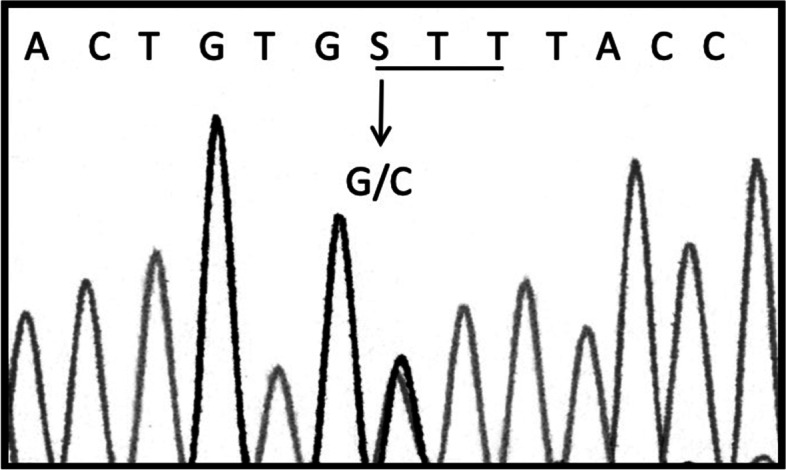
Sequencing electropherogram of exon 1. A heterozygous G → C transition is shown where a S is reported, corresponding to nucleotide 406 of the BMP-15 coding sequence (NCBI Reference Sequence NM_005448). The variant replaces a conserved valine at position 136 with a leucine residue (V136L)

No genetic abnormalities of GDF-9, and FMR1 premutation were found in our group, while in one patient a mutation, potentially pathogenetic, of BMP-15 gene, not described in literature (Fig. 1) was found. Automated sequence showed a heterozygous 406 G > C transition (second exon), changing valine to leucine at position 136 of the protein (V136L) (Genebank AH007120). This variant was found in one only patient of the study group and in none of the controls. In the same patient we found also the polymorphism c.308A > G that automated sequence revealed as a heterozygous missense substitution determining the N103S modification (serine in the place of asparagine). This patient was admitted to our Department in September 2014. She had menarche at age of 11, followed by oligo-amenorreha (> 30 days between two menstrual cycles) for about 8 months and consecutive secondary amenorrhoea. She underwent various hormonal treatment with recovery of mentrual cycles under estro-progestinic therapies. In 2004 at age of 18 she developed hypergonadotropism (FSH 107 mU/ml, LH 63 mU/ml), hypoestrogenism and secondary amenorrhoea, diagnostic for POI.

The patient had also chronic autoimmune thyroiditis with euthyroidism and she had a family history for thyreopathy (mother with chronic autoimmune thyroiditis) and diabetes mellitus (paternal grandparents). Osteodensitometry showed normal values at femoral site and at lumbar spine. Molecular analysis for X-fragile syndrome and cytogenetic analysis of kariotype did not show any abnormality (46 XX).

### Predictive analysis

**Fig. 2:**
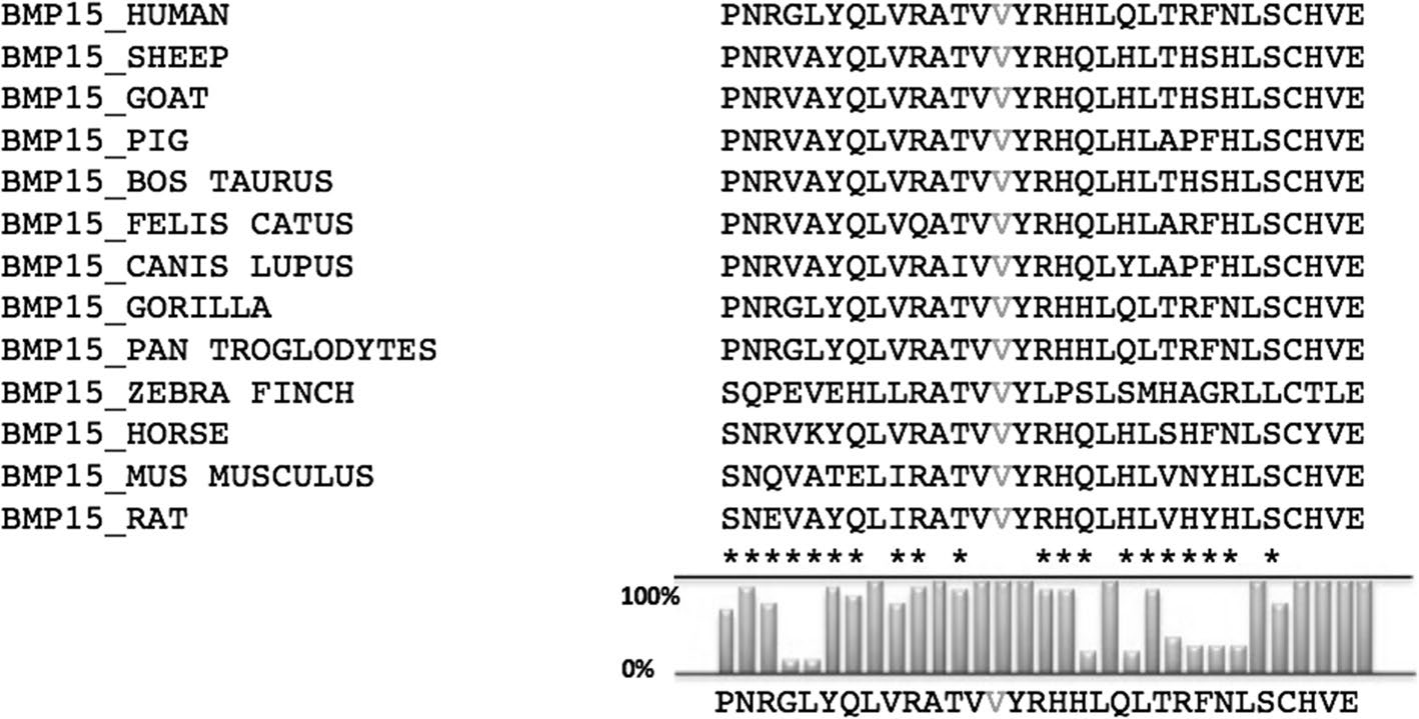
Alignment of the amino acid sequence of part of the BMP-15 propeptide region from several mammalian species with its human homologue BMP-15. The valine affected by the mutation is highly conserved

Poliphen and SIFT programs used predicted that the variant BMP15-V136L was deleterious to the structure with a score of 0.826/1 (sensitivity: 0,84; specificity: 0,93) and function of the encoded protein (Table 2). At position 136 of the protein valine is highly conserved among species (Fig. 2).

**Table 2:** In silico analysis of BPMP-15 mutant

Software	p.V136L	
PolyPhen	*Possibly damaging*	*score* 0,83
SIFT	*Tolerated*	*score* 0,42

### Functional analysis of BMP-15 variant using luciferase reporter assay

**Fig. 3:**
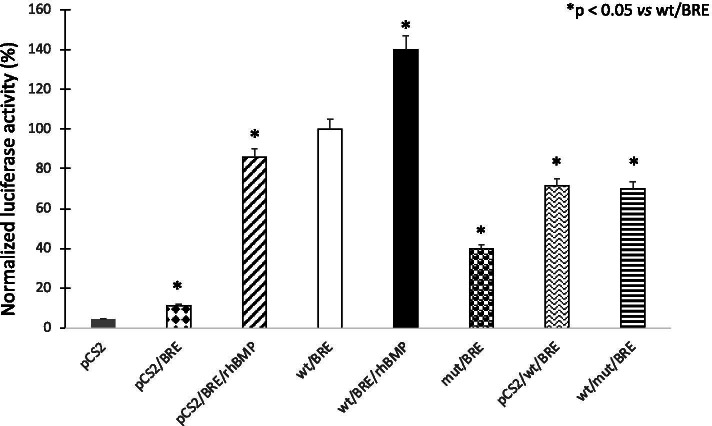
The transcriptional activity of the V136L variant located in the propeptide region of BMP-15 was investigated in the granulosa derived COV434 cells transiently transfected with empty vector ± 100 ng recombinant human BMP-15 (pCS2 ± BMP-15) or wild type human BMP-15 expressing vector (WT) or the V136L variant vectors obtained by directed-mutagenesis of residues under positive selection. Results are expressed as the mean (± SD) of three independent experiments. Differences between means were analyzed with Student’s t-test comparing each condition to WT (*, *p*-value < 0.05)

We evaluated the functional significance of the BMP15-V136L in comparison with that of the wtBMP-15 by means of a reporter assay based on their ability to stimulate a BMP inducible promoter driving the luciferase expression. COV434 cells co-transfectated with BRE-luciferase reporter construct showed specific activation of the BMP-signaling pathway when stimulated with 10 ng/ml of the exogenous recombinant human BMP-4 agonist and transiently transfected with the wt-BMP15 expressing construct or with pcS2 +  + empty vector (Fig. 3).

The luciferase activity of the wtBMP15 and pcS2 +  + cotransfected with BRE-luc and stimulated with exogenous recombinant human BMP-4 agonist resulted equally to 100%.

In contrast, COV434 cells co-transfected with BMP15-V136L variant and BRE-luc showed a significant decrease (-60% vs. wtBMP15) of luciferase activity.

When the COV434 cells were cotransfected with BMP15-V136L variant and an equal concentration of wtBMP15, to reproduce in vitro the heterozygous status in the proband, the luciferase activity was partially restore of the 30% respected to the cells transfected with the wtBMP15 alone (Fig. 3).

## Discussion

POI is a primary ovarian defect characterized by primary or secondary amenorrhea before the age of 40 with hypergonadotropism and hypoestrogenism.

All the women of our study presented secondary amenorrhea before 40 years old. 2 patients presented primary amenorrhea. The mean age of menopause is 26.6 years. FSH value was over 25 mUI/ml in the whole group.

Characteristically, almost half (44%) of patients who underwent transvaginal pelvic ultrasonography had poor follicle assets with few millimetric follicles or small ovaries’ size.

Three patients (17%) had a family history of POI.

Screening for autoimmunity was performed, measuring anti-ovarian antibodies, anti-thyroid antibodies (anti-thyroperoxydase (TPO) and anti-thyroglobulin (TG) antibodies), anti-adrenal antibodies, anti-gastric parietal cells antibodies and antitissue transglutaminase (tTG) antibodies. 1 patient presented anti-ovarian autoantibodies; 7 patients had anti-TG antibodies and 3 of them presented anti-TPO autoantibodies too; 1 woman had anti-gastric parietal cells autoantibodies.

Autoimmunity etiology characterized about 5% of total POI cases. Prevalence of association between POI and many autoimmune diseases was estimated in 20–55% [20, 21]. POI is frequently associated with autoimmune thyroiditis (20%) and until 24% of women with POI present anti-TPO autoantibodies [8]. In our group, nine patients (50%) presented with a personal history of one or more autoimmune diseases: two patients presented Graves’ disease, seven patients had autoimmune thyroiditis. One patient had celiac disease and one had an history of diffused giant urticaria. Finally in our study, we found an association between POI and autoimmune thyroiditis of 38.8% and between POI and autoimmune diseases of 50%. In total 16.7% of patients presented anti-TPO autoantibodies.

In order to evaluate the effect of hypoestrogenism on bone, 15/18 women underwent femoral and lumbar spine osteodensitometry and 2 of them (13%) showed reduced bone density. In this study we realized genetic analysis of all the 18 patients in order to identify the karyotype, possible X chromosomal abnormalities, mutations/polymorphism of BMP15 and GDF-9 genes, and FMR1 premutation.

The analysis of the kariotype of the whole cohort of women did not show any abnormality (46XX).

There weren’t any abnormalities of the X chromosome such as X monosomy (Turner’s syndrome), X deletions, chromosome X translocations.

No genetic abnormalities of GDF-9, and FMR1 premutation were found in our group.

In one patient with secondary amenorrhea and diagnosis of POI at age of 18 years we found a pathogenetic mutation of BMP-15 gene, not previously described in literature (Fig. 1). The new variant involves second exon of BMP15 gene and causes heterozygous substitution c.406G > C, determining the V136L modification (leucine in the place of valine). The mutation is predicted to be possibly damaging by *PolyPhen-2* software. The in vitro functional study showed a significant decrease of luciferase activity when COV434 cells were cotransfected with BMP15-V136L variant in the homozygous and in the heterozygous status, confirming the pathogenetic role of the mutation in development of POI.

As described previously, BMP15 is an oocyte-specific growth/differetiation factor and his role is critical to ovarian reserve determination. It promotes follicle development, granulosa cell mitosis, ovulation rate physiology and modulating granulosa cell sensitivity to FSH [22]. Prevalence of BMP15 gene mutations in POI is variable between 1.5% and 12% among studies [19, 22, 23]. Di Pasquale reported the first mutation of BMP15 gene in two sisters with hypergonadotropic ovarian failure characterized by primary amenorrhea and ovarian dysgenesis [24]. Subsequently, they have extended the genetic screening and identified new BMP15 nonsynonymous variants affecting 4.2% of 46XX idiopathic POI women [23]. In a previous study we reported a heterozygous missense substitution c.538G > A determining the A180T modification in the pro-region of the protein in one patient who developed secondary amenorrhea at the age of 31 years [9]. Mayer et al. described a family with two sisters with POI, harboring a compound heterozygous deletion of each allele of the BMP15 gene, transmitted in a recessive mode, as a “knockout-like” effect with complete lack of mature BMP15 and consequentely precocious follicle degeneration [25]. A number of other mutations and rare deletions in this gene have been described in women with POI [19, 22, 26], confirming his role as a major determinant of ovulation quota and dominant follicle selection in mammals. Several other genes have been reported to contribute to the genetic etiology of POI, including FMR1, FSHR, NOBOX, PGRMC1, GDF9, FOXO3, FIGLA, NR5A1, FOXL2, STAG3, SYCE1, HFM1, NUP107, MCM8, MCM9, MSH5, MSH4, KHDRBS1, EIF4ENIF1, PSMC3IP, CLPP and abnormal Y chromosome [3]. These genes are involved in various processes, including primordial germ cell development, DNA repair and meiosis, oocyte transcription and translational control during folliculogenesis, granulosa cell development and mitochondrial function [27]. The availability of the next-generation sequencing technology has resulted in a growing list of genes causing POI in the last years, revealing that it is a genetically complex disease [28, 29].

## Conclusions

In summary, this study provides new information on POI genetics. We identified a novel mutation, c.406 G > C p.V136L in BMP15 gene that causes a reduction in the activity of the mature protein. This mutation was not found in any controls introduced in the study. This expands the mutation spectrum related to POI and contributes to the understanding of the molecular basis of the disease.

## Data Availability

Not applicable.

## Abbreviations

POI: Premature ovarian insufficiency
COV434: Human ovarian granulosa tumour cells 434
DEXA: Dual Energy X-ray Absorptiometry
SIFT: Sorting Intolerant From Tolerant
PolyPhen-2: Polymorphism Phenotyping v2
RLU: Relative light units

## References

[1] Goswami D, Conway GS (2007). Premature ovarian failure. Horm Res.

[2] Coulam CB, Adamson SC, Annegers JF (1986). Incidence of premature ovarian failure. Obstet Gynecol.

[3] Qin Y, Jiao X, Simpson JL, Chen ZJ (2015). Genetics of primary ovarian insufficiency: new developments and opportunities. Hum Reprod Update.

[4] Cramer DW, Xu H, Harlow BL (1995). Family history as a predictor of early menopause. Fertil Steril.

[5] De Vos M, Devroey P, Fauser BC (2010). Primary ovarian insufficiency. Lancet.

[6] Daan NMP, Muka T, Koster MPH, van Lennep JER, Lambalk CB (2016). Cardiovascular risk in women with premature ovarian insufficiency compared to premenopausal women at middle age. J Clin Endocrinol Metab.

[7] Podfigurna-Stopa A, Czyzyk A, Grymowicz M, Smolarczyk R, Katulski K, Czajkowski K (2016). Premature ovarian insufficiency: the context of long-term effects. J Endocrinol Invest.

[8] Persani L, Rossetti R, Cacciatore C, Bonomia M (2009). Primary Ovarian Insufficiency: X chromosome defects and autoimmunity. J Autoimmun.

[9] Ferrarini E, Russo L, Fruzzetti F, Agretti P, De Marco G, Dimida A (2013). Clinical characteristics and genetic analysis in women with premature ovarian insufficiency. Maturitas.

[10] Moore RK, Erickson GF, Shimasaki S (2004). Are BMP-15 and GDF-9 primary determinants of ovulation quota in mammals?. Trends Endocrinol Metab.

[11] Otsuka F, Yao Z, Lee T, Yamamoto S, Erickson GF, Shimasaki S (2000). Bone morphogenetic protein-15. Identification of target cells and biological functions. J Biol Chem.

[12] Moore RK, Shimasaki S (2005). Molecular biology and physiological role of the oocyte factor, BMP-15. Mol Cell Endocrinol.

[13] Webber L, Davies M, Anderson R, Bartlett J, Braat D, Cartwright B (2016). ESHRE Guideline: management of women with premature ovarian insufficienc. Hum Reprod.

[14] Ng PC, Henikoff S (2006). Predicting the effects of amino acid substitutions on protein function. Annu Rev Genomics Hum Genet.

[15] Kumar P, Henikoff S, Ng PC (2009). Predicting the effects of coding non-synonymous variants on protein function using the SIFT algorithm. Nat Protoc.

[16] Adzhubei IA, Schmidt S, Peshkin L, Ramensky VE, Gerasimova A, Bork P (2010). A method and server for predicting damaging missense mutations. Nat Methods.

[17] Auclair S, Rossetti R, Meslin C, Monestier O, Di Pasquale E, Pascal G (2013). Positive selection in bone morphogenetic protein 15 targets a natural mutation associated with primary ovarian insufficiency in human. PLoS ONE.

[18] Korchynskyi O, ten Dijke P (2002). Identification and functional characterization of distinct critically important bone morphogenetic protein-specific response elements in the Id1 promoter. J Biol Chem.

[19] Rossetti R, Di Pasquale E, Marozzi A, Bione S, Toniolo D, Grammatico P (2009). BMP15 mutations associated with primary ovarian insufficiency cause a defective production of bioactive protein. Hum Mutat.

[20] Hoek A, Schoemaker J, Drexhage HA (1997). Premature ovarian failure and ovarian autoimmunity. Endocr Rev.

[21] Bakalov VK, Anasti JN, Calis KA, Vanderhoof VH, Premkumar A, Chen S (2005). Autoimmune oophoritis as a mechanism of follicular dysfunction in women with 46 XX spontaneous premature ovarian failure. Fertil Steril.

[22] Laissue P, Christin-Maitre S, Touraine P, Kuttenn F, Ritvos O, Aittomaki K (2006). Mutations and sequence variants in GDF9 and BMP15 in patients with premature ovarian failure. Eur J Endocrinol.

[23] Di Pasquale E, Rossetti R, Marozzi A, Bodega B, Borgato S, Cavallo L (2006). Identification of new variants of human BMP15 gene in a large cohort of women with premature ovarian failure. J Clin Endocrinol Metab.

[24] Di Pasquale E, Beck-Peccoz P, Persani L (2004). Hypergonadotropic ovarian failure associated with an inherited mutation of human bone morphogenetic protein-15 (BMP15) gene. Am J Hum Genet.

[25] Mayer A, Fouquet B, Pugeat M, Misrahi M (2017). BMP15 “knockout-like” effect in familial premature ovarian insufficiency with persistent ovarian reserve. Clin Genet.

[26] Dixit H, Rao LK, Padmalatha VV, Kanakavalli M, Deenadayal M, Gupta N (2006). Missense mutations in the BMP15 gene are associated with ovarian failure. Hum Genet.

[27] Moriwaki M, Moore B, Mosbruger T, Neklason DW, Yandell M, Jorde LB (2017). Mutations are associated with primary ovarian insufficiency in women. J Endocr Soc.

[28] Jolly A (2019). Exome sequencing of a primary ovarian insufficiency cohort reveals common molecular etiologies for a spectrum of disease. J Clin Endocrinol Metab.

[29] Lee Y, Kim C, Park Y, Pyun JA, Kwack K (2016). Next generation sequencing identifies abnormal Y chromosome and candidate causal variants in premature ovarian failure patients. Genomics.

